# Intraoperative post-annuloplasty three-dimensional valve analysis does not predict recurrent ischemic mitral regurgitation

**DOI:** 10.1186/s13019-020-01138-7

**Published:** 2020-07-02

**Authors:** Frank Meijerink, Inez J. Wijdh-den Hamer, Wobbe Bouma, Alison M. Pouch, Ahmed H. Aly, Eric K. Lai, Thomas J. Eperjesi, Michael A. Acker, Paul A. Yushkevich, Judy Hung, Massimo A. Mariani, Kamal R. Khabbaz, Thomas G. Gleason, Feroze Mahmood, Joseph H. Gorman, Robert C. Gorman

**Affiliations:** 1 Gorman Cardiovascular Research Group, University of Pennsylvania, Philadelphia, PA USA; 2 Department of Cardiothoracic Surgery, University of Groningen, University Medical Center Groningen, Groningen, the Netherlands; 3 Department of Surgery, University of Pennsylvania, Philadelphia, PA USA; 4 Department of Radiology, University of Pennsylvania, Philadelphia, PA USA; 5 Department of Cardiology, Massachusetts General Hospital, Harvard Medical School, Boston, MA USA; 6 Department of Cardiothoracic Surgery, Beth Israel Deaconess Medical Center, Harvard Medical School, Boston, MA USA; 7 Department of Cardiothoracic Surgery, University of Pittsburgh, Pittsburgh, PA USA; 8 Department of Anesthesia, Beth Israel Deaconess Medical Center, Harvard Medical School, Boston, MA USA

**Keywords:** Ischemia ▪ mitral regurgitation ▪ mitral valve repair ▪ three-dimensional echocardiography

## Abstract

**Background:**

High ischemic mitral regurgitation (IMR) recurrence rates continue to plague IMR repair with undersized ring annuloplasty. We have previously shown that pre-repair three-dimensional echocardiography (3DE) analysis is highly predictive of IMR recurrence. The objective of this study was to determine the quantitative change in 3DE annular and leaflet tethering parameters immediately after repair and to determine if intraoperative post-repair 3DE parameters would be able to predict IMR recurrence 6 months after repair.

**Methods:**

Intraoperative pre- and post-repair transesophageal real-time 3DE was performed in 35 patients undergoing undersized ring annuloplasty for IMR. An advanced modeling algorhythm was used to assess 3D annular geometry and regional leaflet tethering. IMR recurrence (≥ grade 2) was assessed with transthoracic echocardiography 6 months after repair.

**Results:**

Annuloplasty significantly reduced septolateral diameter, commissural width, annular area, and tethering volume and significantly increased all segmental tethering angles (except A2). Intraoperative post-repair annular geometry and leaflet tethering did not differ significantly between patients with recurrent IMR (*n* = 9) and patients with non-recurrent IMR (*n* = 26). No intraoperative post-repair predictors of IMR recurrence could be identified.

**Conclusions:**

Undersized ring annuloplasty changes mitral geometry acutely, exacerbates leaflet tethering, and generally fixes IMR acutely, but it does not always fix the delicate underlying chronic problem of continued left ventricular dilatation and remodeling. This may explain why pre-repair 3D valve geometry (which reflects chronic left ventricular remodeling) is highly predictive of recurrent IMR, whereas immediate post-repair 3D valve geometry (which does not completely reflect chronic left ventricular remodeling anymore) is not.

## Background

Despite the general belief that undersized ring annuloplasty is the preferred surgical treatment strategy for all patients with ischemic mitral regurgitation (IMR), the durability of undersized ring annuloplasty is limited by the fact that recurrent IMR develops in 30% or more of patients in a few months after surgery [1, 2]. IMR recurrence adversely affects outcome [3] and is predominantly related to continued left ventricular (LV) remodeling and worsening of tethering caused by undersized annuloplasty. The Cardiothoracic Surgical Network (CTSN) trial showed that the rate of recurrence after undersized annuloplasty for moderate and severe IMR might be up to 58.8% after 2 years [4]. Using three-dimensional echocardiography (3DE) and advanced valve modeling algorithms, we showed that P3 preoperative segmental leaflet tethering is a strong predictor of recurrent IMR 6 months after undersized annuloplasty [5]. In a subsequent study we showed a much higher predictive value of 3DE over two-dimensional echocardiography (2DE) for recurrent IMR [6]. In addition to preoperative 3DE predictors we are also in need of intraoperative (immediate post-repair) 3DE predictors that can guide patient-specific intraoperative surgical decision-making.

The literature on mitral valve (MV) geometry directly after annuloplasty is relatively sparse. 3DE valve analysis might give us more accurate insights into the quantitative effect of annuloplasty on mitral valve geometry. 2DE studies have shown that undersized annuloplasty may exacerbate leaflet tethering and that this may be a possible mechanism of recurrent IMR [7–9].

We hypothesized that the degree of intraoperative post-repair mitral leaflet tethering would determine the risk of IMR recurrence after undersized ring annuloplasty. We have previously published the pre-repair 3DE and 2DE results of 50 patients with IMR who underwent repair [5, 6], and to determine the predictive value of immediate post-repair 3DE we performed additional 3DE analyses in this same group of 50 patients.

## Methods

This study was approved by the Institutional Review Boards of the University of Pennsylvania, the University of Pittsburgh, and the Beth Israel Deaconess Medical Center. Written informed consent was obtained from all patients.

### Patients and image acquisition

Fifty patients with severe IMR underwent MV repair with an undersized annuloplasty ring. Pre- and immediate post-repair TEE was performed in all patients. Fifteen patients had intraoperative post-repair 3DE images of insufficient quality for modeling and were excluded. The remaining 35 patients were included for analysis. Table 1 shows an overview of the patient characteristics. Ring type selection was at the discretion of the surgeon.

**Table 1:** Preoperative and intraoperative patient characteristics

Variable^a^	Normal	Non-recurrent	Recurrent
	(*n* = 21)	IMR (*n* = 26)	IMR (*n* = 9)
Age, years	66.1 ± 14.4	67.5 ± 9.7	65.9 ± 6.0
Female	8 (38)	8 (31)	3 (33)
Body mass index, kg/m^2^	32.2 ± 8.0	28.4 ± 4.3	28.2 ± 4.2
Medical history			
Hypertension	11 (52)	19 (73)	7 (78)
Diabetes	6 (29)	12 (46)	4 (44)
Renal insufficiency	3 (14)	6 (23)	1 (11)
Atrial fibrillation	2 (10)	11 (42)	3 (33)
Stroke	2 (10)	2 (8)	1 (11)
Previous PCI	3 (14)	7 (27)	4 (44)
Previous CABG	2 (10)	3 (12)	4 (44)
NYHA class, 1-4 scale	2.4 ± 0.8	2.3 ± 1.0	2.8 ± 0.4
IMR grade, 0-4 scale	0.3 ± 0.5	3.0 ± 0.7^c^	3.0 ± 0.8^d^
Inferior wall motion abnormality	0 (0)	23 (88)	9 (100)
Left ventricular			
End-diastolic diameter, cm	4.7 ± 0.8	5.7 ± 0.9^c^	6.1 ± 0.9^d^
End-systolic diameter, cm	3.2 ± 0.8	4.6 ± 1.0^c^	5.1 ± 1.4^d^
Ejection fraction, %	65.2 ± 10.1	36.0 ± 15.7^c^	31.9 ± 11.6^d^
Annuloplasty ring			
Profile 3D ring^e^	−	12 (46)	8 (89)
CE Physio II ring^f^	−	7 (27)	1 (11)
CG Future band^e^	−	6 (23)	0 (0)
St. Jude tailor flexible ring^g^	−	1 (4)	0 (0)
Ring size, mm	−	28.9 ± 1.7	28.7 ± 1.4
Concomitant procedures			
CABG	6 (29)	20 (77)^c^	6 (67)
Aortic valve replacement	14 (67)	2 (8)^c^	0 (0)^d^
Tricuspid valve repair	0 (0)	1 (4)	1 (11)
Atrial maze	0 (0)	5 (19)	0 (0)
Atrial septal defect closure	1 (5)	0 (0)	0 (0)

*3D* three-dimensional, *CABG* coronary artery bypass grafting, *IMR* ischemic mitral regurgitation, *NYHA* New York Heart Association, *PCI* percutaneous coronary intervention

^a^Data are presented as mean±standard deviation or number (%)

^b^*P* < 0.05 recurrent vs non-recurrent

^c^*P* < 0.05 non-recurrent vs normal

^d^*P* < 0.05 recurrent vs normal

^e^Medtronic, Minneapolis, MN

^f^Carpentier-Edwards, Irvine, CA

^g^St. Jude Medical, St. Paul, MN

Two-dimensional (2D) transthoracic echocardiography was performed preoperatively and six months after repair. Images were acquired through a transthoracic apical four chamber view. Severity of IMR was determined semi quantitatively with color Doppler by assessing the area of the regurgitant jet as a percentage of left atrial area in the apical four chamber view. The following grading scale was used: grade 0, no IMR; grade 1, less than 20%; grade 2, 20 to 40%; grade 3, 40 to 60%; and grade 4, more than 60% ^8^. Recurrent IMR 6 months after repair was defined as IMR ≥ grade 2.

Pre-repair real-time 3D TEE images were obtained after induction of anesthesia and prior to sternotomy. Post-repair images were obtained after MV repair and weaning off cardiopulmonary bypass. TEE images were obtained with a systolic blood pressure of at least 100 mmHg. IMR grade was 0 (no/trace) or 1 (only mild) in all patients after repair. TEE images were also obtained for 21 patients with normal mitral valves and LV function who underwent cardiac surgery for indications other than MV disease. Images were acquired through a mid-esophageal view using a Philips IE33 (Philips Medical, Andover, MA) ultrasound system equipped with a 2- to 7-MHz X7-2t TEE matrix transducer. During 4 cardiac cycles the images were acquired with a volumetric frame rate of 17–30 Hz and an imaging depth of 12–16 cm. This way a full volume real-time 3DE data set could be obtained.

### Image segmentation and annular and leaflet modeling

The full volume 3D data set was exported to an Echo-View 5.4 (TomTec Imaging Systems, Munich, Germany) software workstation. All analyses were performed in midsystole. Techniques of annular and leaflet segmentation have been described previously [5, 6]. The Cartesian (x,y,z) coordinates of each data point of the 500–1000 data point cloud for each mitral valve were exported to Matlab (The Mathworks, Inc., Natick, MA) for quantitative analysis. We have previously shown that our 3DE modeling technique is characterized by a very small interoperator and intraoperator variability [10].

Determination or calculation of septolateral diameter, intercommissural width, transverse diameter, annular area, annular circumference, tethering area, tethering volume, tethering index, posterior- and anterior tethering angle, and segmental (mean) tethering angles were described previously [5, 6].

Mitral valve tethering index was calculated by dividing mitral valve tethering volume by mitral annular area. Tethering volume is strongly influenced by annular size and to correct for annular geometry we introduced the mitral valve tethering index [11].

### Statistical analysis

All calculations were performed using IBM SPSS Statistics 23.0 (IBM Corporation, Chicago, IL). Continuous variables were expressed as mean ± standard deviation. Categorical variables were expressed as percentages. Comparisons between groups were performed using Pearson χ^2^ test or Fisher exact test (two-sided) as appropriate for categorical variables and the independent samples t-test or Mann-Whitney U test (two-sided) as appropriate for continuous variables. Within-group comparisons were performed using the paired samples t-test for continuous variables. Statistical significance was established at *P* < 0.05.

## Results

### Patient characteristics

A total of 9 patients (26%) experienced recurrent IMR 6 months after undersized annuloplasty. Based on these findings patients were divided in recurrent and non-recurrent IMR groups. Preoperative and intraoperative patient characteristics are presented in Table 1. As a reference data from 21 patients with normal mitral valves and normal LV function were included in Table 1. None of the baseline variables were significantly different between the recurrent and non-recurrent groups.

### Annular and leaflet geometry

**Fig. 1:**
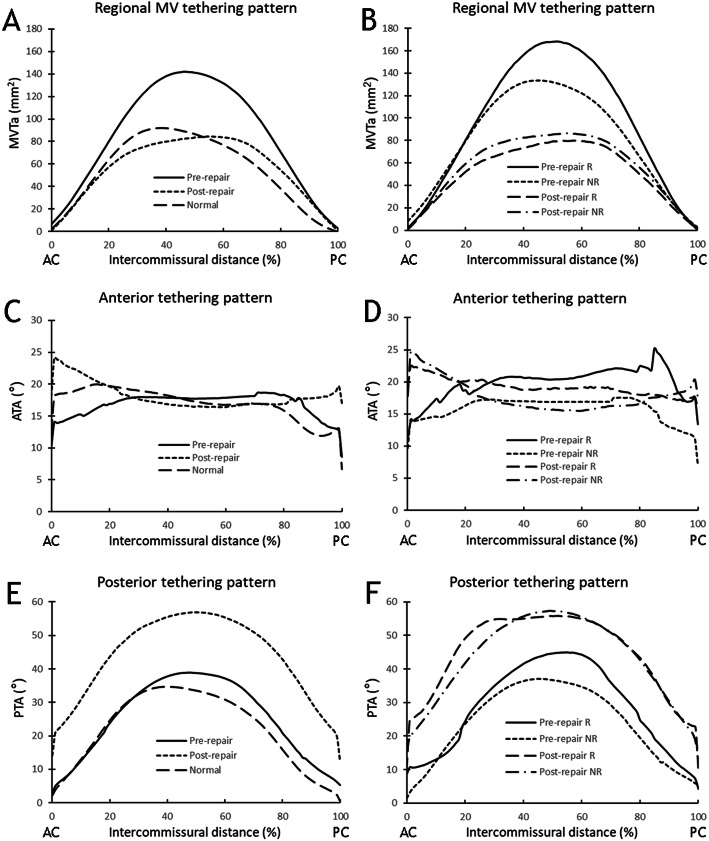
Pre- and post-repair regional mitral valve tethering patterns plotted against the distance travelled between the anterior commissure (AC) and posterior commissure (PC), (**a**, **b**) Regional mitral valve tethering area (MVTa) distribution pre- and post-repair. (**c**, **d**) Regional anterior tethering angle (ATA) distribution pre- and post-repair. (**e**, **f**) Regional posterior tethering angle (PTA) distribution pre- and post-repair. MVTa, ATA and PTA are plotted as a function of intercommissural distance, expressed as a percentage of the distance traveled from the AC. The positions of the AC and PC are 0 and 100%, respectively. NR = non-recurrrent; R = recurrent

In Table 2 an overview is given of all annular and leaflet tethering measurements, before and after ring annuloplasty. All annular parameters were significantly reduced after annuloplasty (*P* < 0.05 for all variables). MV tethering volume was significantly reduced (*P* < 0.05), tethering index did not change significantly (*P* = 0.96). All segmental tethering angles significantly increased after annuloplasty, except for the A2 tethering angle. The posterior tethering angles (P1-P3) changed most (> 20°). Figure 1 shows plots of the regional mitral valve tethering patterns against the distance traveled between the anterior and posterior commissure.

**Table 2:** Pre- and postoperative three-dimensional echocardiographic annular and leaflet tethering variables

		Preoperative			Postoperative		
Variable^a^	Normal (*n* = 21)	Non-recurrent IMR (*n* = 26)	Recurrent IMR (*n* = 9)	Total (*n* = 35)	Non-recurrent IMR (*n* =26)	Recurrent IMR (*n* = 9)	Total (*n* = 35)
Septolateral diameter, mm	28.7 ± 5.1	31.5 ± 4.0	31.2 ± 5.6	31.4 ± 4.4^d^	22.7 ± 4.2	21.2 ± 2.5	22.3 ± 3.9^c,e^
Commissural width, mm	31.4 ± 3.2	32.4 ± 5.5	31.3 ± 7.4	32.1 ± 6.0	29.3 ± 3.3	28.2 ± 2.4	29.0 ± 3.1^c,e^
Mitral annular area, mm^2^	786 ± 155	949 ± 232	923 ± 300	942 ± 247^d^	582 ± 158	536 ± 71	571 ± 141^c,e^
Mitral valve tethering volume, mm^3^	1771 ± 689	2748 ± 1631	3589 ± 1782	2964 ± 1686^d^	1879 ± 1211	1602 ± 537	1808 ± 1078^c^
Mitral valve tethering index	2.25 ± 0.70	2.80 ± 1.23	3.68 ± 1.13	3.03 ± 1.25^d^	3.03 ± 1.26	2.98 ± 0.84	3.01 ± 1.15^e^
Segmental tethering angle, °							
A1	18.4 ± 9.2	19.4 ± 8.6	24.2 ± 7.8	20.6 ± 8.6	27.2 ± 11.4	26.6 ± 12.1	27.1 ± 11.4^c,e^
A2	15.0 ± 8.2	26.5 ± 11.2	32.5 ± 12.3	28.1 ± 11.6^d^	26.9 ± 12.5	31.9 ± 12.3	28.2 ± 12.4^e^
A3	9.5 ± 6.4	13.8 ± 12.1	22.8 ± 10.4	16.2 ± 12.2^d^	24.1 ± 10.0	29.2 ± 6.1	25.4 ± 9.4^c,e^
P1	16.5 ± 8.5	23.3 ± 12.9	28.5 ± 6.2	24.6 ± 11.7^d^	43.2 ± 13.6	46.5 ± 9.4	44.1 ± 12.6^c,e^
P2	17.9 ± 12.0	27.4 ± 17.4	42.5 ± 9.1^b^	31.3 ± 16.9^d^	53.8 ± 11.6	51.7 ± 12.9	53.3 ± 11.8^c,e^
P3	14.0 ± 7.6	18.4 ± 14.1	33.8 ± 4.2^b^	22.3 ± 14.0^d^	43.7 ± 11.8	42.0 ± 11.6	43.3 ± 11.6^c,e^

*IMR* ischemic mitral regurgitation

^a^Data are presented as mean ± standard deviation

^b^*P* < 0.05 recurrent vs non-recurrent

^c^*P* < 0.05 post-repair vs pre-repair

^d^*P* < 0.05 pre-repair vs normal

^e^*P* < 0.05 post-repair vs normal

**Fig. 2:**
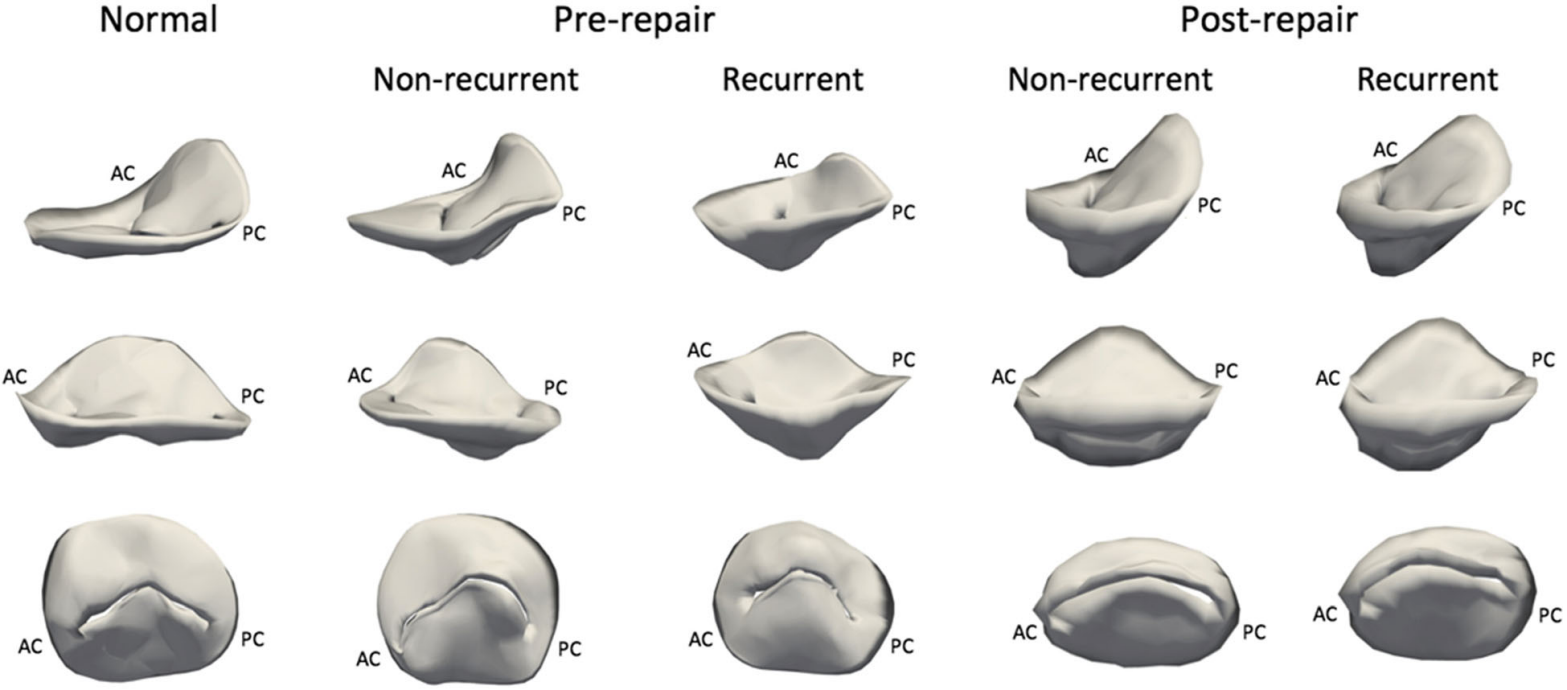
Three-dimensional reconstructed models of a representative normal mitral valve and representative pre- and post-repair mitral valves that will and will not develop recurrent IMR after undersized ring annuloplasty: (top row) oblique commissure-to- commissure view, (middle row) oblique septolateral view, (bottom row) left ventricular view. AC = anterior commissure; PC = posterior commissure

A comparison between the non-recurrent and recurrent IMR subgroups was made in Table 2. For the pre-operative annular measurements there was no significant difference between the recurrent and non-recurrent groups. For the tethering variables, P2 and P3 tethering angles were significantly different (*P* < 0.05). All postoperative annular and tethering variables were not significantly different between recurrent and non-recurrent groups. Figure 2 shows representative 3D reconstructions of normal and pre- and post-repair mitral valves that will or will not develop recurrent IMR 6 months after repair.

## Discussion

Undersized annuloplasty is generally still considered the preferred surgical treatment strategy for IMR [1–3, 12, 13]. Despite successful initial elimination of IMR, its durability is limited by high recurrence rates. In this study a recurrence rate of 26% was observed at 6 months. Earlier studies reported recurrence in approximately one third of the patients within 12 months after the procedure [1, 2, 13]. Recurrence adversely affects outcome [3] and therefore preoperative identification of patients who are at risk of IMR recurrence after undersized annuloplasty repair is of paramount importance to optimize the results of mitral valve surgery for IMR. Our previous work showed that 3DE valve analysis is highly predictive of recurrent IMR, with P3 tethering angle as a very strong independent predictor for recurrent IMR [5]. We also showed that a 3DE-based predictive model was stronger than a 2DE based predictive model in the same population [6]. In addition to the need to be able to preoperatively identify patients who are at risk of IMR recurrence after repair, there is a clear need for powerful intraoperative (immediate post-repair) 3DE (geometric) predictors that can identify patients at risk of IMR recurrence and guide patient-specific intraoperative surgical decision-making. The current study was designed to provide insights into this relatively unknown area.

This post-repair study clearly shows the immediate effect of undersized annuloplasty on 3D annular and leaflet geometry. Although MV tethering volume was significantly reduced after repair, which can be explained by the fact that the mitral annular area is extensively reduced by annuloplasty, leaflet tethering increases significantly in nearly all segments. An important finding in this study is the very high and significant increase in posterior leaflet tethering. This is in line with the results of previous 2DE studies and has been proposed as a possible mechanism of recurrent IMR [7, 8]. Zhu et al. investigated pre- and post-repair mitral leaflet configurations in a 2DE study [7]. This study included 31 patients who underwent undersized annuloplasty for IMR and post-repair echocardiography 2 week to 2 months after surgery. Results showed that post-operative tethering was predominant for the posterior leaflet and that patients with recurrence had a significantly higher posterior leaflet angle than patients without recurrence [7]. The predominant role of posterior tethering in IMR recurrence is best explained by the fact that the anterior annulus is relatively fixed and therefore less influenced. Although our study shows increased post-repair leaflet tethering, the extent of overall anterior and posterior leaflet tethering immediately after repair was similar among patient with recurrent and non-recurrent IMR in this study, which is insufficient evidence to explain it as a causative mechanism of IMR recurrence. In time, however, this may be a moving target with continued LV remodelling and dilatation which continue to exacerbate leaflet tethering and which may render patients prone to IMR recurrence. Hung et al. indeed showed that recurrence was associated with a higher LV diameter and LV sphericity index [2]. The fact that this is a moving target might be a good explanation for our results.

Pre-repair leaflet tethering is basically a reflection of subvalvular remodeling in IMR and is a predictor of IMR recurrence in several studies [5, 14, 15]. Immediately after annuloplasty, the annular and leaflet tethering geometry changes, but subvalvular geometry does not. This process starts after repair and revascularization, and so it is not evident in the immediate post-repair phase. This may explain why immediate post-repair tethering measurements are not predictive for recurrent IMR. Additional quantitative 3DE analysis and modeling of the subvalvular apparatus (tendinal chords, papillary muscles and LV) should be performed in future studies both immediately after repair and several months after repair to further elucidate this concept.

The one and two year outcomes of the CTSN trial showed that there was no difference in LV diameter after mitral valve repair versus replacement, nor in a composite endpoint of major adverse cardiac or cerebrovascular related events, functional status or quality of life [4, 16]. IMR recurrence rates were higher for the repair group. Though it should be noted that in the repair-group, LV end-systolic volume index for the non-recurrent IMR group was significantly lower compared to the recurrent IMR group. These findings also indicate that advanced LV remodeling and dilatation play a role in IMR recurrence.

A substudy of the CTSN trial by Capoulade et al focused on a relatively new parameter, the LV end-systolic diameter to ring size ratio, which appeared to be a significant predictor for IMR recurrence [17]. This “LV-ring mismatch” may be a valuable addition to the various predictors for IMR recurrence. Future treatment modalities should also focus on subvalvular targets in order to minimize “LV-ring mismatch” and the risk of IMR recurrence. We think that 3DE modeling may be a very useful and powerful addition to this combined valvular-subvalvular concept, which could guide future patient-specific surgical planning and decision making.

Our previous studies have shown that pre-repair 3DE mitral valve modeling is highly predictive of IMR recurrence and that its predictive value is much higher than that of 2DE mitral parameters [5, 6]. The current 3DE mitral valve modeling study has two important additional clinical messages: (1) undersized annuloplasty severely exacerbates 3DE posterior leaflet tethering, and (2) immediate post-repair 3DE geometry and leaflet tethering do not predict IMR recurrence and are at this point of no additional value in guiding patient-specific intraoperative decision making.

There were several limitations in this study. (1) The number of patients was relatively small (*n* = 35) and follow-up was relatively short (6 months). Fifteen of the original 50 patients had intraoperative post-repair 3DE images of insufficient quality for modeling and were excluded. (2) The segmentation and modeling methodology of 3D TEE images requires time-consuming off-line analysis. Therefore, work is in progress to develop and validate an automated segmentation technique that will allow image processing and mitral leaflet segmentation in minutes rather than hours [18, 19]. This tool can potentially be used in the operating room to guide surgical decision making for IMR. (3) The end point was an echocardiographic measurement of IMR recurrence, not a clinical outcome such as survival. However, there is strong evidence correlating IMR with reduced survival [20, 21]. (4) Recurrent IMR is measured semi-quantitatively with transthoracic echocardiography color Doppler, where the jet area is expressed as a percentage of the LA area. Alternative assessments of IMR (regurgitant volume and effective regurgitant orifice area) were unfortunately not available in this study.

## Conclusions

Undersized ring annuloplasty changes mitral geometry acutely, exacerbates leaflet tethering, and generally fixes IMR acutely, but it does not always fix the delicate underlying chronic problem of continued LV dilatation and remodeling. This may explain why pre-repair 3D valve geometry (which reflects chronic LV remodeling) is highly predictive of recurrent IMR, whereas immediate post-repair 3D valve geometry (which does not completely reflect chronic LV remodeling anymore) is not.

## Data Availability

Please contact the corresponding author for data requests.

## Abbreviations

2D(E): Two-Dimensional (Echocardiography)
3D(E): Three-Dimensional (Echocardiography)
AC: Anterior Commissure
ATA: Anterior Tethering Angle
CABG: Coronary Artery Bypass Grafting
CTSN: Cardiothoracic Surgical Network
IMR: Ischemic Mitral Regurgitation
LV: Left Ventricle
MV: Mitral Valve
MVTa: Mitral Valve Tethering Area
NYHA: New York Heart Association
PC: Posterior Commissure
PCI: Percutaneous Coronary Intervention
PTA: Posterior Tethering Angle
TEE: Transesophageal Echocardiography
